# Supplementary material for: Are carbon emissions trading and green financial instruments synergistic? -Comprehensive quantitative research based on content analysis
Source: PLoS One. 2024 Mar 7;19(3):e0298601. doi: 10.1371/journal.pone.0298601 (PMC10919601; doi:10.1371/journal.pone.0298601)
Supplement: S1 Data — (ZIP) [file pone.0298601.s001.zip › Data/地级市Z_F工作报告.zip!/╣≤╓▌/┴∙┼╠╦«╩╨/└┤╫╘╣1⁄2╓┌║┼í╛┬φ┐╦╩2╛▌═°í┐_2012.docx]

各位代表：

　　现在，我代表市人民政府，向大会报告工作，请予审议，并请各位政协委员和列席的同志提出意见。

　　过去五年政府工作回顾

　　第六届市人民政府任期的五年，是六盘水发展史上极不平凡的五年，是经济社会发展取得显著成绩的五年，是在全面建设小康社会道路上阔步前进的五年。五年来，在省委、省政府和市委的坚强领导下，在市人大、市政协的监督、支持和帮助下，我们坚持以邓小平理论、“三个代表”重要思想为指导，深入贯彻落实科学发展观，紧紧依靠全市各族人民，克服了国际金融危机、特大自然灾害的影响，扭住发展不放松，凝心聚力抓落实，圆满完成了第六届人民政府各项工作任务，全市经济社会步入了又好又快、更好更快发展的轨道。

　　（一）经济持续快速增长，综合实力跨越攀升。2011年，全市生产总值完成613.4亿元，是2006年的2.4倍，年均增长15%；人均生产总值突破3000美元。财政总收入和一般预算收入分别完成128.46亿元和70.84亿元，是2006年的3.14倍和4.2倍，年均增长25.71%和33.27%。全社会固定资产投资五年累计完成1506.84亿元，是上一个五年的3.5倍。社会消费品零售总额完成157.61亿元，年均增长18.7%。城镇居民人均可支配收入和农民人均纯收入分别达16370元和4437元，年均实际增长7.9%和11.1%。2011年末金融机构存、贷款余额分别为577.02亿元和416.97亿元，是2006年末的2.54倍和2.49倍。

　　（二）结构调整力度加大，发展方式加快转变。传统支柱产业进一步做大，原煤产能达到8000万吨，电力装机容量达到700万千瓦，钢铁产能达到500万吨，水泥产能达到500万吨。老鹰山煤化工一期工程建成投产。新兴产业不断涌现，西南天地煤机制造、明阳风电设备制造等一批机械制造项目开工建设。经济开发区及产业园区建设全面展开。农业结构调整取得新成效，特色农产品基地规模不断扩大，建成省级、市级龙头企业61户。商贸、物流、旅游、文化、房地产等服务业加快发展。“十一五”规划确定的节能减排目标全面完成，单位生产总值能耗和主要污染物排放总量控制在省下达的指标范围内。

　　（三）基础设施明显改善，瓶颈制约逐步缓解。交通建设取得突破性进展，六沾铁路复线建设加快推进，长昆铁路客运专线开工建设；镇胜高速公路境内段建成通车，水盘高速公路、六镇高速公路、六六高速公路、杭瑞高速公路境内段开工建设；新建等级公路447公里、农村公路4502公里、运煤公路500公里；六盘水月照机场开工建设；建成了毛口、野钟、中寨航运码头。水利建设力度加大，黔中水利枢纽一期工程和白河沟、双桥、鱼洞坝、旧院、卡河等一批重点水利工程开工建设，完成病险水库治理31座，解决了55.13万人的饮水困难。电力基础设施建设不断加强，建成500千伏、220千伏、110千伏变电站34座，城乡用电保障能力不断提高。生态建设和环境保护力度进一步加大，完成营造林面积131万亩，治理水土流失面积288平方公里、石漠化面积306平方公里，森林覆盖率提高到38%，年均增长1个百分点以上。广播、电视、通信和互联网快速发展，在城市实现了全面普及，在农村实现了广泛覆盖。

　　（四）城镇建设快速推进，城市功能日益完善。完成了第三轮城市总体规划修编，市中心建成区面积扩展到60平方公里。凉都大道、人民路全线贯通，水黄路入城段改造工程如期完成，红桥大道建设加快，市中心城区路网逐步形成。乌蒙山国家地质公园、凉都森林公园、明湖湿地公园、麒麟山文化公园、环城林带建设和水城河综合治理加快推进，城市人均公共绿地面积达到8平方米，绿化覆盖率达到35.3%。城市公交、供气、供水等公共服务设施保障能力明显提升，一批垃圾中转站、垃圾填埋场、污水处理厂、公共停车场建成使用。深入开展“双创双建”活动，城市形象不断改善，城市品位不断提升，先后荣获全国十佳避暑旅游城市、全国十佳绿色环保标志城市等称号。全市城镇化率提高到38%，步入了城镇化加速发展的新阶段。

　　（五）改革开放力度加大，发展活力不断增强。大力深化国有企业改革，水钢与首钢实现资产重组，盘江精煤股份公司主业整体上市；基本完成了驻市省属大企业企业办社会职能移交。推行国有资本经营预算制度。投融资体制改革步伐加快，组建了六盘水市商业银行，运用BT、BOT等融资模式建设了一批基础设施；财政体制改革深入推进，预算管理不断加强，国库集中收付制度全面建立。农村综合改革稳步推进。医药卫生、集体林权、文化体制等改革取得新突破。对外开放水平进一步提高，区域经济合作和对口帮扶不断深入，招商引资五年累计到位资金522亿元，是上一个五年的3.94倍。民营经济加快发展，占全市生产总值的比重从30%提高到40%。

　　（六）社会事业全面进步，社会建设得到加强。各级各类教育全面发展，城乡办学条件明显改善，学前三年幼儿入园率、初中阶段毛入学率、高中阶段毛入学率分别从2006年的30.05%、92%、34.43%提高到2011年的57%、111.72%、53.27%，“两基”工作通过国家督导评估检查，六盘水师专实现“专升本”。公共文化服务体系不断完善，群众文化体育活动丰富多彩，竞技体育取得新突破，非物质文化遗产保护工作卓有成效。城乡公共卫生体系建设步伐加快，医疗卫生服务水平明显提高。新型农村合作医疗参合率从74.01%提高到97.52%，连续五年位居全省前列。人口出生率从12.9‰下降到12.6‰，出生人口性别比从125.68下降到108.12。双拥共建富有成效，军政军民团结更加巩固，荣获“全国双拥模范城”称号。妇女、儿童、老龄、残疾人等事业健康发展，科技、民族、宗教、外事、侨务、对台、档案、气象、人防、共青团、工会、红十字等工作取得新成绩。

　　（七）民生问题有效解决，发展成果人民共享。积极扩大就业再就业，累计新增城镇就业12.36万人，转移农村劳动力29.69万人。启动了省级创业型城市创建工作。城乡低保实现动态管理下的应保尽保，保障对象分别达到 7.21万人、42.89万人。基本养老、基本医疗、失业、工伤和生育保险覆盖面不断扩大。完成农村危房改造8.59万户、“美好家园”建设1.22万户，建设廉租房31020套、经济适用住房7368套、公租房2200套。国有重点煤矿采煤沉陷区综合治理工程基本完工。高度重视和关心城乡困难群众生产生活，发放临时价格补贴1.09亿元、生活用煤补贴4.75亿元。加大扶贫开发力度，减少农村贫困人口26.91万人。安全生产形势总体平稳，煤炭百万吨死亡率、道路交通万车死亡率、亿元GDP死亡率和10万从业人员死亡率明显下降。深入开展“平安凉都”创建活动，摆脱了连续四年安全感指数测评全省排名挂末的被动局面。信访和维稳工作进一步加强，全市社会大局保持稳定。

　　五年来，我们始终坚持把政府自身建设作为做好各项工作的重要保障。自觉接受人大的法律监督、工作监督和政协的民主监督，认真办理人大代表议案、建议和政协提案。加强与民主党派、工商联、无党派人士和人民团体的联系，认真听取各界人士的意见和建议。“五五”普法圆满完成，依法治市得到加强。市、县政府机构改革全面完成。深入推进政务公开，扎实开展“创先争优”、“三个建设年”、“四帮四促”等活动，大力推进机关效能建设，行政效能进一步提高。坚持不懈地开展反腐败斗争，切实加大审计、监察力度，大力纠正部门和行业不正之风，查处了一批腐败分子。

　　2011年，全市经济社会继续保持了良好的发展势头，实现了“十二五”良好开局。全市生产总值增长16.6%；财政总收入和一般预算收入分别增长20.68%和43.66%；全社会固定资产投资增长66.5%；社会消费品零售总额增长20%；城镇居民人均可支配收入和农民人均纯收入分别增长17.6%和23.2%。

　　各位代表！回顾五年的奋斗历程和生动实践，我们深刻体会到：一是必须把解放思想作为首要法宝。思想的领先是最重要的领先，观念的滞后是最致命的滞后。要始终坚持与时俱进、自强不息、兼收并蓄、海纳百川的思想境界，用全局意识、世界眼光、战略思维谋划发展，使我们的思想观念、行为方式和工作方法不断适应形势的发展变化。二是必须把加快发展作为首要任务。发展是解决所有问题的关键。快发展小困难，慢发展大困难，不发展难上难。要始终坚持加快发展不动摇、不懈怠、不折腾，在科学发展观的指引下，做到好中求快、快中保好，又快又好、更快更好。三是必须把保障改善民生作为首要目标。民生连着民心，民心凝聚民力。要始终把人民群众满意不满意、答应不答应、高兴不高兴作为做好政府工作的出发点和落脚点，察民情、解民忧、顺民怨、纾民困，切实做到发展为了人民，发展依靠人民，发展成果由人民共享。四是必须坚持把改革开放作为加快发展的首要动力。改革开放是促进科学发展、实现富民强市的动力源泉。要始终以深化改革突破体制障碍，以扩大开放增创发展优势，实现更高层次和更高水平的发展。五是必须把真抓实干作为首要准则。干字当头，敢字为先，拼字求胜。要始终增强发展的信心和决心，以踏石留印、抓铁有痕的精神，脚踏实地，崇尚实干，不务虚功，以一流的作风创造一流的业绩。

　　各位代表！过去五年取得的成绩来之不易。这是省委、省政府坚强领导、科学引领、亲切关怀和省直各部门支持帮助的结果，是历届领导班子薪火相传、继往开来、接力奋斗的结果，是全市各族人民同心同德、锐意进取、不懈奋斗的结果。在此，我代表市人民政府，向在过去五年中为六盘水市经济社会发展作出艰苦努力和付出辛勤劳动的全市广大工人、农民、知识分子、干部，向各民主党派、工商联、无党派爱国人士和各人民团体，向驻市人民解放军、武警官兵、公安干警、民兵和预备役人员，向所有关心和支持我市改革开放和现代化建设的各界人士，表示衷心的感谢并致以崇高的敬意！

　　在肯定成绩的同时，我们也清醒地看到，我市经济社会发展还存在诸多不容忽视的问题，主要是：第一，发展速度慢，经济总量小仍是我市的主要问题。虽然纵向比我市发展有进步，但横向比差距在拉大，我市的综合发展竞争力排名在全国地级市中5年就后退了几十位，在省内的经济综合实力排位也从上游滑到中游。第二，瓶颈制约突出。交通、水利等基础设施欠账较多，城乡功能性基础设施不完善，不能适应经济社会发展的需要。第三，发展的协调性不强。城乡发展不平衡，城乡居民收入差距一直在4:1左右，高于全国、全省平均水平；教育、文化等社会事业发展还比较滞后，社会管理比较薄弱，人民群众安全感不高，安全生产形势仍然严峻。第四，经济对资源的依存度较高。产业结构单一，发展方式粗放，经济增长的资源环境代价过大，节能减排、生态建设和环境保护的任务相当艰巨。第五，改革开放力度不大。市场机制不完善，市场配置资源能力较弱，对外开放水平不高，投资环境还不尽人意。第六，政府自身建设仍不适应新要求。政府职能转变还不到位，社会管理和公共服务比较薄弱；工作落实不到位，办事效率低，不作为乱作为及以权谋私等现象还不同程度存在。对于这些问题，我们将采取有力措施，认真加以解决。

　　对今后五年政府工作的建议

　　第七届市人民政府任期的五年，是我市加速发展的黄金期、加快转型的关键期、推动跨越的攻坚期和全面建设小康社会的决战期。东部产业向中西部地区转移进程加快，资本要素加速流动，我市发展空间无限广阔；国务院出台了《关于进一步促进贵州经济社会又好又快发展的若干意见》，我们迎来了千载难逢的发展机遇；经过30多年的改革开放，我市经济社会发展的基础条件不断改善，发展后劲不断增强，后发优势逐步显现。六盘水谋求大发展、推进大转型、实现大跨越，其势已成，其时已至，发展的潮流势不可挡。我们一定要树立信心、提振精神、坚定意志、攻坚克难，在新一轮发展中赢得主动、赢得优势、赢得未来！

　　今后五年，政府工作的总体要求是：高举中国特色社会主义伟大旗帜，以邓小平理论和“三个代表”重要思想为指导，深入贯彻落实科学发展观，坚持“两加一推”主基调，全面贯彻落实市第六次党代会精神，按照“全党抓经济、重点抓工业、首要抓园区、核心抓招商、突破抓转型、跨越抓项目”的思路，大力实施工业强市、城镇化带动和农业现代化提升三大战略，大力实施国民经济和社会发展第十二个五年规划，以贯彻落实国务院《关于进一步促进贵州经济社会又好又快发展的若干意见》为总抓手，深化改革，扩大开放，增加就业，保障民生，推进生态文明、环境友好、资源节约、和谐社会建设，打造现代特色都市，努力建设经济发达、政治昌明、文化繁荣、社会稳定、人民幸福的实力六盘水、魅力六盘水、活力六盘水、文明六盘水、幸福六盘水，为与全省全国同步实现全面小康打下坚实基础。

　　今后五年，全市经济社会发展的主要目标是：到2016年，全市生产总值达到1600亿元以上；财政总收入、一般预算收入、城镇居民人均可支配收入和农民人均纯收入在2011年的基础上翻一番以上；全社会固定资产投资五年累计完成8000亿元以上。

　　通过五年努力，全市综合经济实力进入全省“第一方阵”，城市综合竞争力进入全国地级城市200名前，全面小康实现程度接近全国平均水平；逐步实现由传统工业城市向现代工业强市转变，农业弱市向特色农业市转变，区域性中心城市向现代特色都市转变；把六盘水建设成为全国循环经济示范城市、统筹城乡综合配套改革试点城市、全国重要的能源基地、资源深加工基地、长江珠江上游重要生态安全屏障和贵州省重要的综合交通枢纽、现代产业聚集区、和谐社会模范区。

　　围绕实现以上目标，要重点做好以下工作：

　　（一）做大经济总量，推动转型升级，努力在建设实力六盘水上实现新突破。充分发挥资源优势，进一步做大二产，做特一产，做优三产。

　　大力发展工业经济。按照“立足煤、依托煤、超越煤、跳出煤”的思路，做大做强传统支柱产业、培育新兴产业、发展关联产业，延长产业链、拓宽产业幅、提高附加值。煤炭产业，加强煤炭资源整装勘查和开发，新建、改造一批安全高效矿井，提高产业集中度，建成2个3000万吨级、5个1000万吨级特大型煤炭企业，矿井数量控制在100个以内。到2016年，煤炭产业产能稳定在1.5亿吨左右，原煤入洗率提高到70%以上，煤炭产业产值达到1150亿元以上。电力产业，建设一批燃煤电厂、矸石电厂、水电站、煤矿瓦斯发电和风力发电，发展企业自备电厂、热电联产车间。寻求在大用户直供电上取得突破。到2016年，电力装机容量达到1500万千瓦以上，电力产业产值达到300亿元以上。钢铁产业，支持首钢水钢（集团）公司升级改造，把其建设成为西部长材精品基地。加快首黔公司300万吨钢材生产项目建设。大力发展钢铁精深加工。到2016年，力争钢产量达到1000万吨以上，钢铁产业产值达到500亿元以上。有色产业，发挥能源优势，建设高技术、高载能园区，重点实施双元铝业扩能改造，积极引进铝、锰精深加工项目。到2016年，铝及铝产品年产能达到30万吨以上，有色产业产值达到100亿元以上。煤化工产业，依托炼焦煤资源，坚持传统煤化工和现代煤化工并举，以六盘水（六枝）煤电化一体化基地、老鹰山煤化工二期工程、首黔公司煤电钢一体化基地等项目为载体，大力发展煤化工产业。着力发展煤制烯烃，统筹推进煤层气、焦炉煤气和过境天然气的综合利用。到2016年，力争达到年产能焦炭1800万吨、煤焦油45万吨、甲醇240万吨、二甲醚40万吨、煤制烯烃80万吨，煤化工产业产值达到700亿元以上。建材产业，围绕固体废弃物的综合利用，建成六枝畅达（二期）、水城豪龙、老鹰山新型干法水泥项目、恒远新型建材（二期）等工程。到2016年，达到年产能水泥2000万吨以上、新型墙体材料1000万立方米以上，建材产业产值达到200亿元以上。装备制造产业，建成西南天地煤矿成套设备制造、明阳风电设备制造、贵州盘江矿山机械公司煤矿机械制造、六枝变频电机车及新型带式输送机生产等项目，把我市打造成西部重要的矿山装备制造基地。到2016年，装备制造产业产值达到300亿元以上。新兴产业，积极培育节能环保、信息技术、生物制药、农副产品加工、食品加工、特色旅游商品加工等新兴产业，促进产业轻型化、高新化、多元化。到2016年，新兴产业产值达到120亿元以上。“八大产业”工业总产值达到3370亿元以上。

　　围绕以上产业发展，一要充分发挥我市资源能源优势，强化园区基础设施建设，以招大商招好商为目标，大力实施“园区企业落地突破工程”，着力推进经济开发区（工业园区）发展，建成一批资源深加工园区、装备制造业园区、高载能园区和物流园区，力争申报成功1个国家级经济开发区。到2016年，经济开发区（工业园区）工业增加值占全市工业增加值比例达到60%以上；二要以全面深入贯彻落实国务院《关于进一步促进贵州经济社会又好又快发展的若干意见》为契机，树立大抓项目、抓大项目的强烈意识，全力实施“大项目突破工程”，五年累计完成工业固定资产投资5000亿元以上；三要依托大企业、服务大企业，深入开展地企合作，推进企业加快技改步伐，强力实施“规模以上企业数量质量突破工程”。到2016年，全市规模以上企业达到1000户以上，打造一批销售收入超100亿元、500亿元的大企业大集团，新增上市公司20户以上。

　　大力发展特色农业。以建设六盘水喀斯特山区特色农业示范区为目标，加快推进设施农业、有机农业、标准农业、观光农业的发展。尤其要在突出特色上下功夫，重点抓好“九大产业”：马铃薯产业，种植面积200万亩左右，总产量300万吨以上，产值40亿元以上；蔬菜产业，建成商品蔬菜基地30万亩以上，总产量60万吨以上，产值9亿元以上；茶叶产业，建成茶叶基地50万亩，产值30亿元以上；油茶产业，建成油茶基地50万亩，产值20亿元以上；猕猴桃产业，建成猕猴桃基地30万亩以上，总产量45万吨以上，产值90亿元以上；核桃产业，建成核桃基地70万亩，总产量11万吨以上，产值22亿元以上；中药材产业，建成中药材基地30万亩，产值9亿元以上；烤烟产业，建成烤烟基地20万亩，总产量50万担以上，产值6亿元以上；畜牧产业，总产值67亿元以上。“九大产业”的总投入累计达到100亿元以上，全部达产后年产值达到300亿元以上。到2016年，将粮经比调整到20:80。

　　围绕实现以上产业发展，要重点建设郎岱、大用、保田、米箩等现代农业产业园区，打造一批年销售收入超亿元的大型生产基地和龙头企业。建立农村土地流转制度，大力推进农田水利基础设施建设，夯实现代农业发展基础。强化科技兴农，提升农业科技支撑。大力实施新型农民培训工程、农村劳动力转移培训工程，造就有文化、懂技术、善经营、会管理的新型农民。

　　大力发展现代服务业。切实抓好以旅游业为重点的生活性服务业。将旅游业作为优化经济结构的战略重点来抓，以2013年省第八届旅游发展大会在我市召开为契机，强力推进旅游基础设施、配套服务设施和旅游接待设施建设，推动旅游与文化、体育、工业、农业等融合发展，大力发展城郊乡村休闲旅游。重点推进北盘江牂牁湖旅游区、凉都森林公园、水城县百车河休闲旅游区、玉舍国家森林公园、盘县坡上草原旅游区、妥乐古银杏景区开发和首钢水钢全国工业旅游示范点建设，推进老鹰山石河景区“三线”遗址建设和六枝煤矿工业旅游开发，着力打造“中国凉都”避暑旅游休闲度假胜地和“乌蒙之巅”、“夜郎故地”、“大三线探秘”、“亚高原运动”等特色旅游品牌，把六盘水打造成贵州西部旅游集散中心和川滇黔结合部重要旅游目的地城市。加强对商贸、餐饮等传统服务业的提升改造。积极发展家政服务、病患陪护、物业管理等家庭服务业和社区服务业。大力发展房地产业，带动相关产业快速发展。切实抓好以物流业为重点的生产性服务业。依托能源资源、产业基地和交通枢纽，加快建设物流节点城市；推动重要工业产品、大宗矿产品、农产品等专业市场建设，发展一批物流园区；加快用现代信息技术改造传统物流业，引进一批物流企业，与优势产业、重点企业联动发展，推动物流产业做大做强。鼓励企业开展服务外包，大力发展法律、会计审计、咨询代理、资产及信用评估、策划设计和生产资料（设备）租赁，促进生产、服务的专业化、产业化。

　　（二）改变城乡面貌，提升城市影响力，努力在建设魅力六盘水上实现新突破。以建设现代特色都市为目标，发挥“中国凉都”品牌效应，高起点、高标准、高质量推进城乡规划建设管理，着力提升城市功能和品位，不断提升城市知名度和美誉度。

　　全力打造生态环保之城。坚持开发与保护、节能与减排、治山与兴水相结合，大力发展循环经济、低碳经济、生态经济，扎实推进资源节约型、环境友好型社会建设。以退耕还林、石漠化综合治理为重点，继续实施天保、珠防等生态工程建设，全面推进城镇公共绿地、环城林带、交通沿线、景区景点绿化建设，到2016年全市森林覆盖率达到45%以上。实施重点流域水污染治理和水生态建设，加强对森林、草地、湿地等生态系统和自然保护区、饮用水源地的保护。提升全社会生态文明理念，积极倡导低碳生产、生活和消费方式，在加快发展中实现青山永驻、碧水长流、天空常蓝。

　　全力打造宜居宜业之城。完成第四轮城市总体规划修编，加强城市控制性详细规划、修建性详细规划编制，实现乡镇、村庄整治规划全覆盖。以城市路网建设为先导，拉大城市框架，加快推进市中心城区向特大城市、盘县红果向大城市、六枝平寨向中等城市的目标迈进，力争到2016年全市城镇化率达到60%。注重城市建筑风格和色彩控制。着力抓好“城中村”和旧城改造，5年内基本消除市中心城区城市棚户区。全面启动城市综合交通、绿化、水系、灯饰“四大系统”建设，切实加强供排水、供电、通讯、供气管网等市政设施及学校、医院、保障性住房、养老服务等功能性设施建设。全面启动国家级卫生、环保、园林、旅游、双拥“五城联创”计划。积极建设“智慧城市”，全面提升城市管理水平。加强社区建设，推进社区服务。强化城市产业支撑，引导产业向城市集聚，为市民提供更多就业岗位。统筹城乡基础设施建设，推动城镇道路、供水、生活垃圾处理等基础设施向农村延伸，公共交通、公共服务向农村拓展。以“四在农家”为载体，大力实施“新农村建设工程”，推进村庄整治和农村危房改造，用5年左右时间，全面完成全市60万户新民居建设和改造，使农村面貌发生天翻地覆的变化。

　　全力打造宜游宜购之城。建成一批功能突出、业态完整、聚集力强的重点商贸设施项目，同步优化城市商业网点结构布局，搞活商贸流通，建成覆盖城乡、辐射周边的商品集散地。着力建设一批现代服务产业聚集区、综合功能配套区和特色商贸区，打造城市中心商圈，吸引国内外著名品牌和知名连锁店入驻。不断丰富消夏文化节内涵，定期举办各类大型商务会展、商业演出、文体活动，以凉都品牌推动旅游，以办节办会增强承载，全面提升城市要素吸纳力、辐射带动力和综合竞争力。

　　（三）坚持改革开放，推进科技创新，努力在建设活力六盘水上实现新突破。抓住国家支持我市开展统筹城乡综合配套改革试点的历史性机遇，进一步解放思想，开拓创新，增创体制机制和对外开放新优势。

　　不断深化重点领域改革。大力实施城乡综合配套改革，着力推进城乡规划、产业布局、基础设施、公共服务一体化，重点在户籍制度、社会保障、土地流转、管理创新等方面加大改革力度，加快构建统筹城乡发展的体制机制。深化国有企业改革，普遍建立现代企业制度。深化投融资体制改革，努力形成投资增长的内在机制。继续深化教育、医疗、文化、财政、收入分配等方面的改革。大力发展民营经济，到2016年，民营经济占全市经济总量的比重达60%以上。加强扩权强县工作，进一步简政放权，建立加快县域经济发展的政策体系，支持六枝奋力攻坚、盘县加速崛起、水城全力突破、钟山率先跨越，确保盘县5年内进入全国百强县行列，水城县2年、六枝特区3年进入全省经济强县行列，钟山区2年内在全省经济强县中的排名进入前5位。

　　大力发展开放型经济。加快构筑以高速公路、快速铁路、城际轻轨、民用航空为重点的现代综合交通运输体系，不断完善经济发展基础条件，聚集各类生产要素。联手推进攀西—六盘水经济区、毕水兴资源富集区规划建设，广泛参与珠三角、长三角、东盟自贸区等区域合作，加快形成对外开放新格局。加强与对口帮扶城市、友好城市、川滇黔十市州的交流，积极开展互访考察和经贸交流活动。全力加强招商引资工作，提高招商引资的质量和效能，力争招商引资实际到位资金年均增长30%。

　　着力建设创新型城市。强化科技创新，加大应用技术研究与开发投入，支持科研院所到我市设立分支机构，建设一批市级以上重点实验室和工程技术中心、企业技术中心，打造一批在煤炭清洁高效利用、装备制造、新材料、新能源、节能环保、现代农业等领域掌握关键技术的知名品牌企业。加强知识产权工作。大力实施创新人才和科技创新团队培育工程。进一步健全柔性引才、稳才机制，创新人才政策，把我市建设成为高端创新人才的聚集地。

　　（四）大力繁荣文化事业，推进文化改革创新，努力在建设文明六盘水上实现新突破。坚持先进文化的前进方向，推动我市文化大发展、大繁荣。

　　实施文明素质提升工程。以创建全国文明城市为目标，广泛开展群众性精神文明创建活动。深入推进文明市民教育，积极开展各种群众性评先选优活动，注重典型引路，示范引导，积极营造文化育人、榜样引人、环境造人的良好氛围。

　　实施特色文化保护工程。加强文化和自然遗产保护与利用，深入挖掘夜郎文化、民族文化、长征文化、工业文化和“三线”文化，在保护中开发利用，在继承中创新发展。大力培养基层文化骨干、民间艺术人才，扶持发展地方民间艺术表演团体。

　　实施文化服务惠民工程。完善覆盖城乡、结构合理、功能健全、实用高效的公共文化服务体系。全面建成市级“七馆四中心”，县级“四馆两中心”，乡镇精神文明活动中心、综合文化站、公共电子阅览室，村级农民文化家园、农家书屋和一批公共文化场所。广泛开展丰富多彩的群众文化活动，推动公共文化资源向社会免费开放，切实保障人民群众的基本文化权益。

　　实施文化产业培育工程。加快培育文化产品市场和要素市场，加强重大文化项目和文化品牌建设，大力发展文化产业。积极打造“中国凉都”避暑文化产业聚集区，大力实施“六大文化产业重点工程”。注重文化人才培养、文化品牌培育和文化企业扶持，建成一批特色文化产业园区和基地，使文化产业逐步成为全市经济发展新的增长点，文化发展主要指标和综合实力进入全省前列。

　　（五）加强社会建设和管理，不断改善人民生活，努力在建设幸福六盘水上实现新突破。更加注重社会建设和管理，着力保障和改善民生，不断提升人民群众的幸福指数。

　　千方百计增加城乡居民收入。坚持富民优先导向，实施“城乡居民收入倍增计划”，加快构建居民收入持续较快增长的多元支撑体系。实施更加积极的就业政策，加强就业培训和就业援助，鼓励自主创业，推动以创业促进就业，力争城镇新增就业16万人。

　　进一步完善社会保障和救助体系。围绕“人人享有基本社会保障”的目标，建立完善社会保险、社会救助、社会福利相互衔接、覆盖城乡的社会保障体系。继续扩大城镇居民社会养老保险和新型农村社会养老保险覆盖面，实现城乡居民社会养老保险全覆盖。全面推进城镇职工、城镇居民基本医疗保险和新型农村合作医疗制度建设。全面提高失业、工伤、生育参保覆盖率。加快推进保障性住房建设，全面改善城乡中低收入群众的住房条件。强化防灾减灾救灾工作。

　　加大扶贫攻坚力度。紧紧抓住国家以乌蒙山片区、滇桂黔石漠化片区等集中连片特殊困难地区为扶贫攻坚主战场的契机，大力实施“减贫摘帽”工程，加大扶贫开发力度，确保六枝、盘县、水城3个国家级贫困县全部实现“减贫摘帽”。

　　统筹推进各项社会事业发展。坚持教育优先发展战略，办好更加规范的学前教育、更加均衡的基础教育、更具特色的职业教育、更高水平的高等教育和更加开放的民办教育，加快建设教育强市。加快完善覆盖城乡的医疗卫生服务体系和健康保障体系，推动城乡医疗卫生资源优化配置，不断满足人民群众基本医疗卫生需求。加快发展体育事业，大力发展竞技体育，深入开展全民健身活动，开工建设一批体育场馆，建成老王山国家生态型多梯度高原运动训练示范基地。加强人口计生工作，统筹解决好人口问题。

　　全面加强和创新社会管理。坚持党委领导，强化政府责任，加大社会协同和公众参与力度，建立科学有效的利益协调、诉求表达、矛盾调处和权益保障机制，坚决纠正损害群众利益的行为。加大公共安全投入，强化安全生产监管，严格责任追究，严肃查处重大安全生产事故背后隐藏的违法违纪案件，健全完善安全生产长效机制，确保反映全市安全生产水平的各项指标接近或超过全国平均水平。认真实施“六五”普法规划，推进依法治市。深入开展“平安凉都”建设，采取更加强硬的措施，严厉打击危害国家安全和社会治安的各种违法犯罪和黑恶势力，坚决扫除黄、赌、毒等社会丑恶现象，保障人民群众生命财产安全，巩固提升群众安全感满意度。加强社会主义精神文明和民主法治建设。支持国防建设和法院、检察院工作。加大妇女儿童发展规划实施力度。切实做好民族、宗教、外事、侨务、老龄、对台、档案、工会、共青团、地方志、红十字、残疾人等工作。

　　2012年工作安排

　　2012年，是新一届政府施政的开局之年，也是继往开来、再创佳绩的关键一年。全市经济社会发展的总基调是“稳中求快”，总要求是“能快则快、又快又好、更快更好”，总目标是“突出特色，做大总量，提速转型，增比进位”。主要预期目标是：生产总值增长18%；全社会固定资产投资增长50%以上；财政总收入和一般预算收入分别增长20%；招商引资到位资金增长110%以上；社会消费品零售总额增长20%以上；城镇居民人均可支配收入和农民人均纯收入分别实际增长15%；人口出生率控制在12.3‰以内；城镇登记失业率控制在4.2%以内；完成省下达的节能减排指标任务。

　　围绕实现上述目标要求，我们将重点抓好以下工作：

　　（一）努力扩大投资规模，拉动经济加速发展。牢固树立抓投资就是抓发展的理念，完成固定资产投资850亿元，力争突破1000亿元。按照“三基本”要求，加快项目前置手续办理，促成项目早日落地建设。实质性新开工800个以上项目（总投资800亿元以上），每季度集中开工200个以上项目。加大产业投资力度，重点投向资源深加工、装备制造，完成工业投资400亿元以上，其中技改投资128亿元以上。研究、吃透国家政策，积极争取上级投入，盘活固定资产，做好土地储备，深化银政合作，做实做细工业、城镇、农业、交通、水利、文化、旅游等项目融资工作，努力争取更大的信贷支持。启动实施“民营企业上市工程”，鼓励有条件的企业上市融资。创新金融产品融资。出台激活民间资金的政策措施，引导本地企业就地投资、转产投资和外流企业回流投资。组建完成市政府金融办。竭力保障项目建设用地需求。强化地方政府重大建设项目征地拆迁安置主体责任，依法、有序、规范、和谐地推进拆迁工作。加大项目前期工作经费投入，加快项目库建设，形成充足的项目储备。加强政府投资项目监管。

　　（二）壮大工业经济规模，加快结构调整步伐。全年完成规模以上工业增加值362亿元，增长26%以上。全力抓好煤炭生产，全年完成原煤产量7150万吨、洗精煤产量2000万吨。开工建设黑塘煤矿、马依煤矿选煤厂等一批重点项目。着力抓好开发区（园区）建设，全年完成基础设施投资60亿元以上，完成产业项目投资320亿元以上，建成标准厂房150万平方米以上。继续加快转型升级，开工建设一批循环经济项目，推进一批技改项目。大力实施民营经济“三年倍增计划”，全面贯彻落实加快民营经济发展的各项政策措施，促进一批人员创业，带动一批人员就业，全年新增微型企业1000户以上。在加快发展工业经济的同时，大力推进服务业发展，全年完成服务业增加值236亿元，增长15%以上。

　　（三）加快城镇规划建设，促进区域协调发展。启动第四轮城市总体规划修编，完成30个乡镇505个村的村庄整治规划编制工作。加快市中心城区城市南北向道路建设，拓展城市发展空间。开工建设150万平方米的城市棚户区改造工程，统筹推进“城中村”和旧城改造。继续加强城市功能性基础设施建设，全面开工建设省第八届旅游发展大会工程、凤凰山城市综合体开发工程和市中心城区集中供热工程。深入开展“整脏治乱”和“双创双建”活动，继续推进水城河综合治理。加强城市周边山体绿化，增加城市绿地面积。开展城市综合交通整治，着力解决城市拥堵问题。严格执行市中心城区土地“五统一”管理，大力筹集城市建设资金。

　　（四）加强“三农”工作，促进农村发展农业增效农民增收。以“四在农家”为载体，搞好房屋亮化、道路硬化、庭院净化、村寨绿化、环境美化，重点在交通沿线、城市周边、乡镇机关所在地及旅游景区建设6万户以上新民居。实施33090户农村危房改造、118个新农村建设试点和水城县蟠龙乡新农村建设“整乡推进”。启动实施“九大产业”推进计划和50万亩农业产业化建设工程，掀起农业结构调整新高潮。着力培育发展龙头企业和农民专业合作经济组织，新增2个以上省级龙头企业、4个以上市级龙头企业。下大力抓好扶贫开发工作，进一步完善和落实扶贫政策措施，拓宽贫困群众增收渠道。实施生态移民工程，完成移民安置4264人。完善结对子帮扶制度，引导城市资金、技术、人才、管理等生产要素向农业和农村流动。大力实施产业化扶贫，实施54个村“整村推进”项目。

　　（五）加快基础设施建设，强力突破发展瓶颈。完成交通投资151亿元。建成六沾铁路复线工程，配合做好长昆高速铁路六盘水境内段建设，加快推进六盘水—安顺城际轨道项目、毕节—水城—兴义铁路前期工作。建成水盘高速公路，完成六镇高速公路主体工程，加快推进六六高速公路、杭瑞高速公路境内段建设，扎实推进盘兴高速公路、六威高速公路前期工作，力争早日开工建设。建成长寨至兴隆、英武至羊场、响水至瓦窑田等二级公路，开工建设郎岱至毛口旅游公路。建成通村油路600公里，新增80个建制村通油路，实现40%建制村通油路。开工建设机场快线。加快六盘水月照机场建设，完成投资6亿元，确保2013年建成通航。开工建设野钟新发旅游码头和毛口旅游码头；完成水利投资56亿元。加快“三位一体”规划水利项目的前期工作，力争“十二五”全部开工建设。配合抓好黔中水利枢纽一期工程建设，加快推进白河沟、双桥、鱼洞坝、旧院、卡河等水库建设，力争开工建设懒龙河、观音岩、出水洞、朱昌河等水库。完成烟水配套工程1.5万亩，治理水土流失面积23.68平方公里，新增、改善和恢复有效灌溉面积4万亩，治理病险水库5座。加快农村饮水安全工程进度，新增解决14万人农村饮水安全问题；推进城乡电网建设与改造，完成投资15亿元，开工建设一批输变电工程，实现农村电网改造全覆盖；加强信息基础设施建设，完成投资5.7亿元。新增80个自然村寨通电话、80个自然村寨通宽带和40个行政村有线广播电视联网延伸覆盖。

　　（六）加强生态文明建设，推进经济社会可持续发展。大力实施以石漠化综合治理为重点的生态修复工程和以林业、水土保持为重点的生态建设工程，力争完成营造林任务25万亩以上，治理石漠化面积68平方公里以上。加强环城林带建设和饮用水源地周边绿化，对重要公路干线沿线两侧25度以上玉米地有计划地开展“退耕还林”。积极推进粉煤灰、煤矸石、废水、废气、废渣、脱硫石膏等废弃物的综合利用。抓好重点领域、重点行业的监管，严格执行节能减排有关政策，全面推进清洁生产。开展全民节能减排行动，完成节能减排任务；加大对重点流域、区域、行业污染治理和农村环境综合整治。加强和完善县（乡）级污水处理设施和垃圾填埋设施建设，启动3万人以上重点建制镇生活污水集中处理设施建设。加强地质灾害综合防治和矿山环境恢复治理。

　　（七）坚持深化改革开放，全面推进招商引资。进一步深化国有企业改革，继续推进水务体制改革，积极稳妥地开展事业单位分类改革，深化医药卫生体制改革。加大招商引资力度，全年完成1600亿元的招商引资任务，实际到位资金达到600亿元以上。以凉都消夏文化节、川滇黔十市州合作与发展峰会等为载体，举办大型招商活动。强化主动服务、上门服务的意识，为企业提供精细化、优质化服务。认真抓好签约项目的跟踪落实，确保签约项目顺利实施。

　　（八）加快推进社会建设，着力保障改善民生。切实做好就业、社保、教育、文化、卫生、计生等各项工作，努力实现包容性增长和共享式发展。一要进一步抓好城乡就业和社会保障。力争实现城镇新增就业6.2万人，培训农村劳动力2.53万人，转移农村劳动力5.56万人。大力推进社会保障体系建设，进一步扩大各类社会保险参保人数。实现城乡居民社会养老保险制度全覆盖，领取养老金人数达27万人。完善城镇居民医保和新型农村合作医疗制度。提高城乡居民最低生活保障标准。继续实施社会救助和保障标准与物价上涨挂钩联动机制，确保城乡困难群众生活水平不降低。建成城镇保障性安居工程住房10062套；新开工建设城镇保障性安居工程住房10642套。二要优先优质发展教育事业。强力推进“4+2”工程，促进城乡教育均衡发展、布局优化。继续实施缓解市中心城区大班额“三年行动计划”，加快解决大班额问题。加快推进市一中、市三中二期工程建设。围绕创建国家示范性高等职业技术院校目标，加快推进省级示范性高等职业技术院校创建工作，开工建设六盘水职业技术学院二期工程。完善六盘水师院一期工程，开工建设六盘水师院二期工程，加强六盘水师院学科和专业建设。4个县、特区、区各建成城区中学1所、小学1所，钟山区加建城区中学1所、小学2所。建成21所乡（镇）幼儿园。建设农村寄宿制学校学生宿舍3万平方米，教师周转宿舍12.58万平方米。实施营养改善计划，实现农村义务教育阶段学校“人人吃午餐”。大力发展中等职业教育、民办教育和特殊教育。统筹抓好老年教育和民族教育。启动钟山水月职业技术教育中心建设。加强师资队伍建设，提高师德水平和教学质量。三要加快发展文化体育事业。开工建设凉都大剧院、市图书馆、市文化馆、市博物馆、市档案馆、市民中心、会展中心、玉舍国家森林公园博物馆和三线遗址博物馆。按照“周周有活动、月月有比赛”的要求，筹划启动一批群众文化体育活动。积极推进老王山国家生态型多梯度高原运动训练示范基地建设。四要大力发展卫生、人口计生等事业。实施卫生基础设施建设工程，加快推进骨干医院建设，启动市人民医院二期工程和创建三甲医院工作，开工建设市精神病医院和市中心血站，加快推进市妇女儿童医院扩建和县级人民医院建设，建成盘县安宁医院，完成5个中心乡（镇）卫生院规范化建设。加快全科医生培养，加强乡村医生队伍建设。加大人口计生工作力度，采取强有力措施，坚决打一场人口计生工作翻身仗，确保摆脱在全省挂末的被动局面。加快科技进步与创新，推进国家煤炭清洁转化检验中心、水钢技术中心建设。

　　（九）加强和创新社会管理，促进社会和谐稳定。扎实开展“信访积案化解攻坚”活动，集中力量化解征地拆迁、移民安置、矿群矛盾、产权纠纷、企业改制、城市公共服务管理等方面的历史遗留问题。继续推进“平安凉都”建设，坚持不懈地开展严打整治斗争，加强天网工程等社会治安综合治理信息化建设，强力推进社区戒毒、社区康复“阳光工程”建设，加大基层基础工作和设施建设力度，力争群众安全感指数继续“升点进位”。以煤矿和道路交通为重点，统筹抓好矿山、建筑、民爆、危化、消防等各行业领域的安全生产工作，确保实现安全事故起数和死亡人数双降。加快食品药品安全保障体系建设，加强食品药品生产经营环节的监管和抽验，严厉打击食品药品领域各类违法违规行为，确保人民群众的食品药品安全。

　　加强政府自身建设

　　全面完成今后五年的各项任务，政府的责任重大、使命光荣。我们将主动接受人大、政协、各民主党派和社会各界的监督，广泛听取各方面的意见、建议，进一步转变政府职能，改进政府工作作风，提高政府办事效率，推动政府管理创新，努力打造人民满意的政府。

　　（一）转变工作作风，提高政府效能。坚持真抓实干、大干快干的务实作风，积极倡导“五加二、白加黑”的奉献精神和“人一之我十之，人十之我百之”的执着奋斗精神，争先创优，激情创业。进一步健全观摩交流、监督检查、跟踪问效、绩效评估等机制，实施奖勤罚懒、重奖严罚，力促公务人员真正把精力集中到想干事上、把本领体现到会干事上、把目标锁定到干成事上。进一步树立敢于尝试、敢于担当、敢于负责的工作作风，善于在挑战中寻求发展机遇、在困境中开创局面，大胆突破不合理的制度和框架束缚，深入研究和用足用活国家发展政策。高规格构建行政电子监察平台、高标准构建公共资源交易平台、高质量构建权力公开运行监管平台、高要求构建问责惩处平台，着力落实限时办结制度、首问负责制度、责任追究制度、奖惩激励制度，努力提高政府办事效率，着力降低群众办事成本。

　　（二）推进职能转变，营造良好的发展环境。结合开展“三个建设年”、“四帮四促”和“三访”活动，深入推进政务公开，全面公开办事制度、办事程序、办理结果，营造公开透明、廉洁高效的政务环境；全力加强政务服务中心建设，做到人员到位、项目到位、授权到位、服务到位，做到行政审批事项“能减则减”，营造便民利民、方便快捷的服务环境；加强法制教育，增强党员干部的法律意识，把加强企业、群众的服务纳入法制化的轨道，主动为经济发展保驾护航，营造依法办事、公正文明的法制环境；健全市场准入机制和管理机制，加强信用体系建设，深入开展整顿和规范市场经济秩序活动，严厉打击不法行为，营造诚实守信、安全和谐的市场环境；坚持以正面宣传，提高舆论引导能力，让全社会共同关心发展、投身发展，营造积极向上、健康发展的舆论环境。

　　（三）狠抓廉政建设，深入开展反腐败斗争。把反腐倡廉建设放在更加突出的位置，坚决贯彻执行中央和省委、市委关于廉洁从政的各项规定，旗帜鲜明地反对腐败。按照“标本兼治、综合治理、惩防并举、注重预防”的方针，进一步完善惩治和预防腐败体系，更加注重从源头上预防和解决腐败问题。深入开展反腐倡廉教育，筑牢拒腐防变思想道德防线。大力提倡艰苦奋斗，坚决制止奢侈浪费。加强廉政监察、效能监察和审计监督，严厉追究失职渎职行为，严肃查处各种违纪违法案件，坚决纠正损害群众利益的不正之风，以反腐倡廉的实际行动赢得人民群众的信任，树立清正廉洁的良好形象。

　　各位代表！我们的任务十分艰巨，我们的前景无限美好。让我们紧密团结在以胡锦涛同志为总书记的党中央周围，在市委的坚强领导和人大、政协的有力监督、支持下，团结和依靠全市各族人民，高举发展、团结、奋斗的旗帜，把思想统一到发展上，把心思集中到发展上，把力量凝聚到发展上，大力实施“三大战略”，打造现代特色都市，为实现全面建设“五个六盘水”的宏伟目标而努力奋斗！
